# Vision Restoration with the PRIMA System in Geographic Atrophy Due to AMD

**DOI:** 10.1056/NEJMoa2501396

**Published:** 2025-10-20

**Authors:** Frank G. Holz, Yannick Le Mer, Mahiul M.K Muqit, Lars-Olof Hattenbach, Andrea Cusumano, Salvatore Grisanti, Laurent Kodjikian, Marco Andrea Pileri, Frederic Matonti, Eric Souied, Boris V. Stanzel, Peter Szurman, Michel Weber, Karl Ulrich Bartz-Schmidt, Nicole Eter, Marie Noelle Delyfer, Jean François Girmens, Koen A. van Overdam, Armin Wolf, Ralf Hornig, Martina Corazzol, Frank Brodie, Lisa Olmos de Koo, Daniel Palanker, José-Alain Sahel

**Affiliations:** 1 Department of Ophthalmology, University of Bonn, Germany; 2 Hôpital Fondation A. de Rothschild, Paris, France; 3 Vitreoretinal Service, Moorfields Eye Hospital, London, Institute of Ophthalmology, University College London, both in the United Kingdom; 4 Department of Ophthalmology, Ludwigshafen Academic Teaching Hospital, Ludwigshafen am Rhein, Germany; 5 Department of Experimental Medicine, University of Rome Tor Vergata, Italy; 6 Department of Ophthalmology, Medical Center Schleswig-Holstein, University of Luebeck, Germany; 7 Service d’Ophtalmologie, Hôpital Universitaire de la Croix-Rousse, Hospices Civils de Lyon, Laboratoire MATEIS, UMR-CNRS 5510, INSA Université Claude Bernard Lyon 1, both in France; 8 Azienda Ospedaliera San Giovanni Addolorata, Rome, Italy; 9 Centre Monticelli Paradis, Marseille, L’Université d’Aix-Marseille, CNRS, INT, Inst Neuroscience Timone, Marseille, both in France; 10 Department of Ophthalmology, Intercommunal Hospital of Créteil and Henri Mondor Hospital, France; 11 Eye Clinic Sulzbach, Knappschaft Hospital Saar, Department of Ophthalmology, University of Bonn, both in Germany; 12 Eye Clinic Sulzbach, Knappschaft Hospital Saar; 13 Nantes University, Nantes University Hospital, Department of Ophthalmology, France; 14 University Eye Hospital Tübingen, Germany; 15 Department of Ophthalmology, University of Münster Medical Center, Germany; 16 Bordeaux University Hospital, France; 17 Hôpital National des 15-20, Inserm-DGOS CIC1423, Paris, France; 18 Department of Ophthalmology, Erasmus Medical Center, Rotterdam, Rotterdam Ophthalmic Institute, Rotterdam, both in the Netherlands; 19 Department of Ophthalmology. University of Ulm, Germany; 20 Science Corporation, Alameda, United States of America; 21 Department of Ophthalmology, University of California San Francisco, San Francisco, United States of America; 22 Department of Ophthalmology, University of Washington, Seattle, United States of America; 23 Department of Ophthalmology, Stanford University, Stanford, United States of America; 24 Department of Ophthalmology, University of Pittsburgh School of Medicine, Pittsburgh, United States of America, Sorbonne Université, Paris, France

## Abstract

**Background:**

Geographic atrophy (GA) due to age-related macular degeneration (AMD) is the leading cause of irreversible blindness and affects over 5 million people globally. Currently, no therapies exist to restore vision to the affected persons. The PRIMA system combines a subretinal photovoltaic implant and near-infrared light-projecting glasses to restore sight to areas of central retinal atrophy.

**Methods:**

In an open-label, baseline-controlled, multicenter, prospective, single-arm clinical study, the vision of participants with GA and a visual acuity of logMAR ≥1.2 was assessed with and without PRIMA glasses at six and twelve months. The primary endpoints were the proportion of participants with improvement in visual acuity (VA) of logMAR 0.2 or more and the number and severity of study-related serious adverse events at 12 months.

**Results:**

Thirty-two of thirty-eight enrolled participants reached the 12-month endpoint. Of the six who did not, three died, one withdrew, and two were unavailable for testing. Twenty-six (81.3%; 95% CI: 63.56–92.79%) had an improved prosthetic VA of at least logMAR 0.2 versus baseline (p<0.001) and in 19 participants, there were 26 serious adverse events. Twenty-one of these events (81%) occurred within the first two months, of which 20 (95%) were resolved within two months of implantation. There was no significant change in peripheral natural VA compared to baseline.

**Conclusions:**

In this study of a limited number of participants, the PRIMA system restored central vision with significant improvements of VA in participants with GA due to AMD. (Funded by Science Corporation, and others; Trial Reg: NCT04676854)

## Background

Age-related macular degeneration (AMD) is the most common cause of incurable blindness in the elderly.^1,2^ Geographic atrophy (GA), an advanced stage of dry AMD, leads to progressive, irreversible death of photoreceptors in the outer retina, causing profound vision loss.^3^ Globally, GA affects approximately 5 million people and in North America it is responsible for ~20% of all cases of legal blindness.^2–4^

The first therapies for GA, designed to slow disease progression using complement inhibitors (pegcetacoplan and avacincaptad pegol), were recently approved in the United States, and require monthly or bimonthly intravitreal injections.^5–7^ However, no approved therapies, investigational approaches, or cell therapies, have shown meaningful visual improvement.^8–12^ In a healthy retina, photoreceptors transduce light into electrical signals which are then processed by the inner retina and transmitted to the brain. Due to a loss of photoreceptors in GA, light is not transduced into electrical signals, leading to an absolute scotoma.^3^ The PRIMA neurostimulation system (hereinafter, PRIMA system) replaces the lost photoreceptors with a photovoltaic implant.^13^

**Figure 1:**
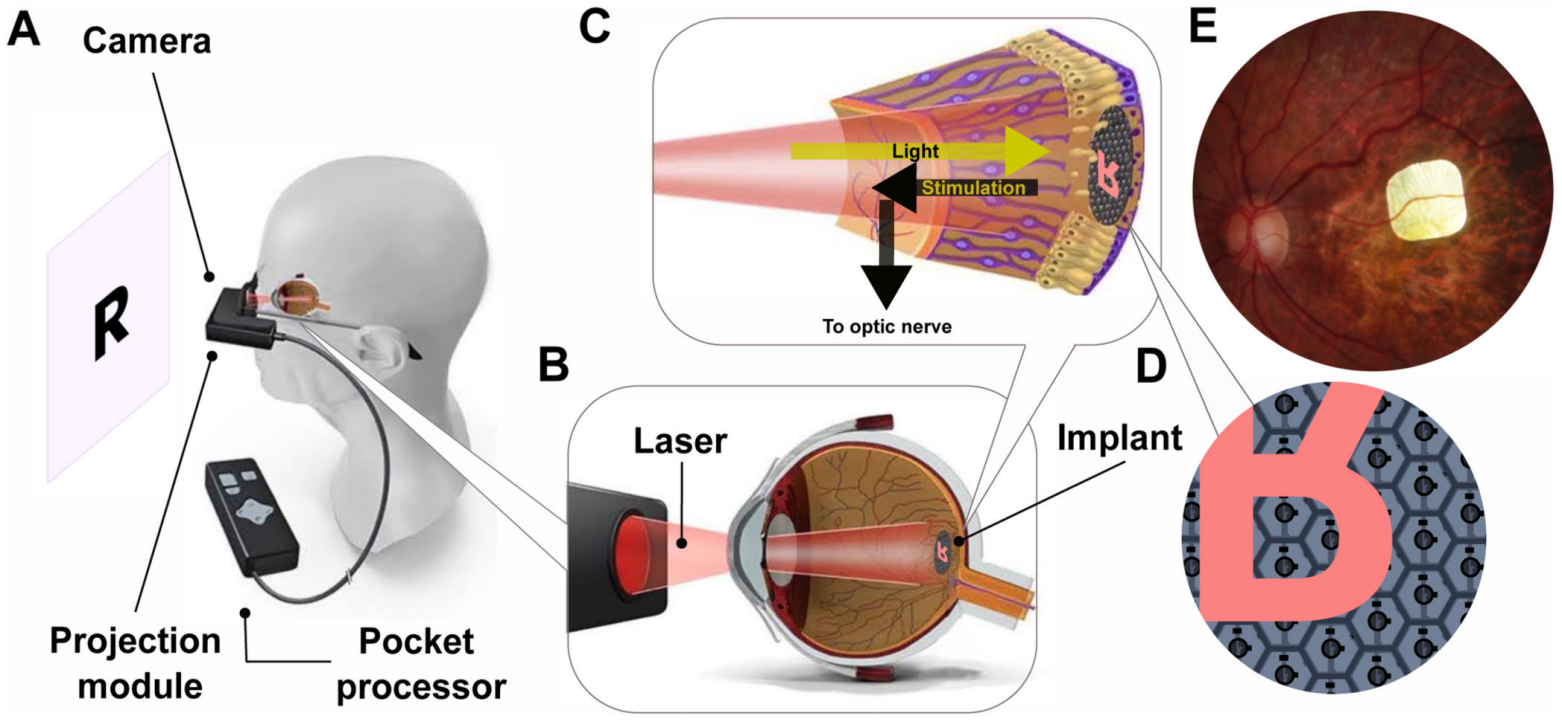
Rendering of the PRIMA system components. (A) The system includes the PRIMA glasses with a camera and projection module, a pocket processor, and the implant. The captured image (e.g., a letter R) is processed and projected onto the implant by NIR (880nm) light. (B) Projection of NIR light onto the implant. (C) The implant, activated by the NIR light, stimulates the remaining nerve cells, which process the information in the retina and then transfer it via the optic nerve to the brain. (D) Photodiodes in each pixel of the subretinal implant convert light into electric currents to stimulate the nearby inner retinal neurons. To utilize flicker fusion for stable perception, the projector operates at a 30Hz frame rate, and perceptual brightness is adjusted by controlling the pulse duration from 0.7 to 9.8ms at peak irradiance of 3.5mW/mm^2^. Each pixel comprises the active electrode in the center, a hexagonal return electrode mesh, and two photodiodes filling the space between the active and the return electrode. (E) Fundus photograph of a participant’s retina with the PRIMA implant. The implant is 2×2mm in size, composed of 100μm pixels.

The PRIMA implant is a 2×2mm wide and 30μm thick crystalline silicon array comprising 378 photovoltaic pixels, each 100μm in size.^14,15^ It is implanted subretinally within the atrophic lesion. A frame-mounted camera on the PRIMA glasses captures images, and projects them, after processing onto the implant, using near-infrared (880nm) light (Figure 1). The implant’s pixels convert near-infrared light into electric pulses to stimulate retinal bipolar cells, restoring the flow of visual information.^14^ Unlike a wired prosthesis, the photovoltaic nature of the implant enables wireless operation combined with a straightforward implantation technique.^16,17^ The lens in the PRIMA glasses is transparent, so participants can perceive natural and prosthetic vision simultaneously.^18^

After extensive pre-clinical testing,^18–20^ a first-in-human clinical trial evaluated the feasibility of the PRIMA system in five participants with GA.^21^ Although the primary endpoint of the feasibility trial was to elicit prosthetic perception, after optimization and training, three participants reliably recognized sequences of letters and had acuity closely matching the 20/420 maximum resolution allowed by the pixel size (100 μm).^15^ At 4 years, these patients could read small fonts, with a mean visual acuity (VA) of 20/135.^21^ The PRIMAvera study was conducted to assess safety and efficacy of the PRIMA system.

## Methods

### Study Design

PRIMAvera is an open-label, baseline-controlled, non-randomized, multicenter, prospective, single-arm, confirmatory clinical study involving 38 participants with GA due to advanced AMD (NCT04676854). The study, conducted at 17 clinical sites across 5 European countries, evaluated the efficacy and safety of the PRIMA system in participants with GA in both eyes and an atrophic patch including the fovea with profound central vision loss in at least one eye. PRIMAvera was designed by Science Corporation with support from its advisory board and approved by local ethics committees. Clinical authors conducted the study, Science Corporation analyzed the data, and the Data Safety and Monitoring Board (DSMB) reviewed it. The corresponding and co-senior authors wrote the manuscript, together with authors from Science Corporation. All authors reviewed and approved the final manuscript for submission. The authors vouch for the accuracy and completeness of data and data analyses, along with the conduct of the trial according to the protocol (available at NEJM.org).

Daniel Palanker and Jose Alain Sahel co-senior authors vouch for the data and the analysis.

Inclusion criteria included: age 60 years or older with a confirmed diagnosis of GA due to AMD in both eyes validated by fundus autofluorescence imaging; VA of logMAR 1.2 (Snellen equivalent 20/320) or worse in the study eye; fovea-involving atrophy greater than the implant size (>2.4mm in diameter) in the study eye.

### Study Endpoints

The primary efficacy endpoint was the proportion of participants (lower bound >50%) achieving a clinically meaningful visual acuity improvement, defined as logMAR ≥0.2 (≥10 letters) on a standard ETDRS chart at 12 months.^15,21–24^ Secondary efficacy endpoints included the proportion of participants with an improvement of VA at 6 months, mean VA improvement at 6 and 12 months, impairment as assessed by Impact-of-Vision-Impairment (IVI) questionnaire at 6 and 12 months, and central visual perception at 12 months.^25,26^

The primary safety endpoint was the number and severity of device- and procedure-related serious adverse events (SAEs) at 12 months. Secondary safety endpoints were the number and severity of all related adverse events (AEs; serious and non-serious), the change in best corrected natural VA (without the PRIMA glasses) compared to baseline, and the percentage of compliant implantations at 4 weeks post-operation. Additional follow-up is planned for up to 36 months.

### Visual Function Assessment

We tested the best corrected VA of the study eye with and without the PRIMA glasses 6 and 12 months after surgery; participants could adjust brightness and zoom level at will. After 12 months, participants answered a survey on home use of PRIMA and their ability to perform various visual tasks.

### Statistical Analysis

Data from all 38 participants were included in the primary analysis. Missing data for primary efficacy endpoints were simulated using multiple imputation models (see Supplementary Appendix) and an additional analysis based on observed data was performed. A binomial test evaluated the proportion of participants achieving an improvement of logMAR ≥0.2, compared to a predefined success threshold of 50%. For the primary endpoint, confidence intervals for binomial testing were computed with the exact method for observed data and Logit method for multiple imputation.

Mean VA improvement from baseline to 12 months was assessed as a secondary endpoint. A sequential gatekeeping approach was tested against a null hypothesis of mean improvement of zero as well as of logMAR 0.2, based on a one-sided α=0.025. For secondary endpoints, exact confidence intervals were used. VA data were analyzed under three conditions: 1) without PRIMA glasses, 2) with PRIMA glasses, and 3) participant’s choice, to reflect home use. The safety cohort analysis was based on observed data without imputation and included all device- and procedure-related SAEs (defined by ISO14155). The DSMB reviewed all device- and procedure-related AEs. All statistical analyses were performed using SAS version 9.4. Surgical procedure, vision training, and additional study methods are detailed in the Supplementary Appendix.

## Results

**Figure 2:**
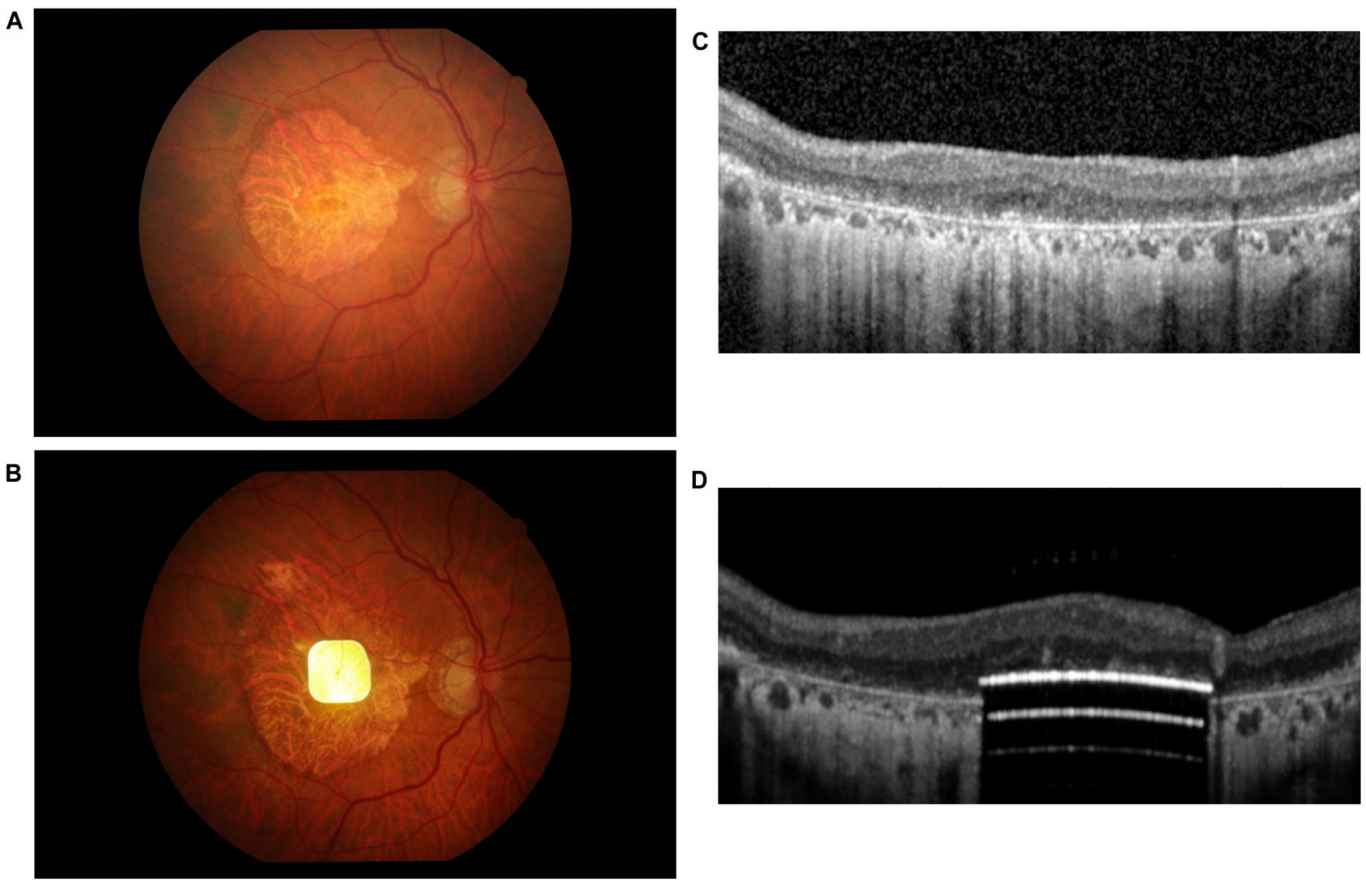
Subretinal implant in a study participant’s eye. This participant had an atrophy size of 21.49 mm^2^ at baseline and was implanted five years after AMD diagnosis. (A) Color fundus photograph demonstrates GA prior to implantation. (B) The same eye with a subretinal implant at 12 months post-implantation. The green arrows indicate the direction of OCT scanning. (C) OCT image of the eye pre-implantation. (D) OCT image demonstrating the implant under the degenerated retina in proximity to the inner nuclear layer.

The PRIMA system was implanted in 38 participants (18 male/20 female) with a mean age of 78.9 years (±6.41 SD) (Table S4). This group is broadly representative of the global GA population (Table S5). Figure 2 shows a representative pre- and post-implantation set of fundus and optical coherence tomography (OCT) images. At 12 months, 32 participants were available for evaluation (Figure S1). Six participants were not assessed due to death (3), withdrawal (1) or unavailability for testing (2) (Table S6).

### Prosthetic vision

Of 32 participants, 30 demonstrated central perception at 12 months. Prosthetic VA (without zoom and image processing) was measured using Landolt C optotypes directly displayed on the PRIMA glasses, without use of the camera (Table S7). The mean VA was logMAR 1.32±0.16 (Snellen 20/417). This average value matches the sampling limit of the 100 µm pixels used by PRIMA (logMAR 1.32, see Supplementary Appendix).

### Efficacy of the system

At 12 months, 81.3% (95% CI: 63.56 to 92.79%; 26/32; p<0.001) of observed participants demonstrated a clinically meaningful improvement in VA (logMAR ≥0.2). Using multiple imputation with pre-specified covariates to account for missing data from six participants, an estimated 79.9% of all participants (95% CI: 65.58 to 94.16%; p<0.001) would have achieved meaningful improvement.

**Figure 3:**
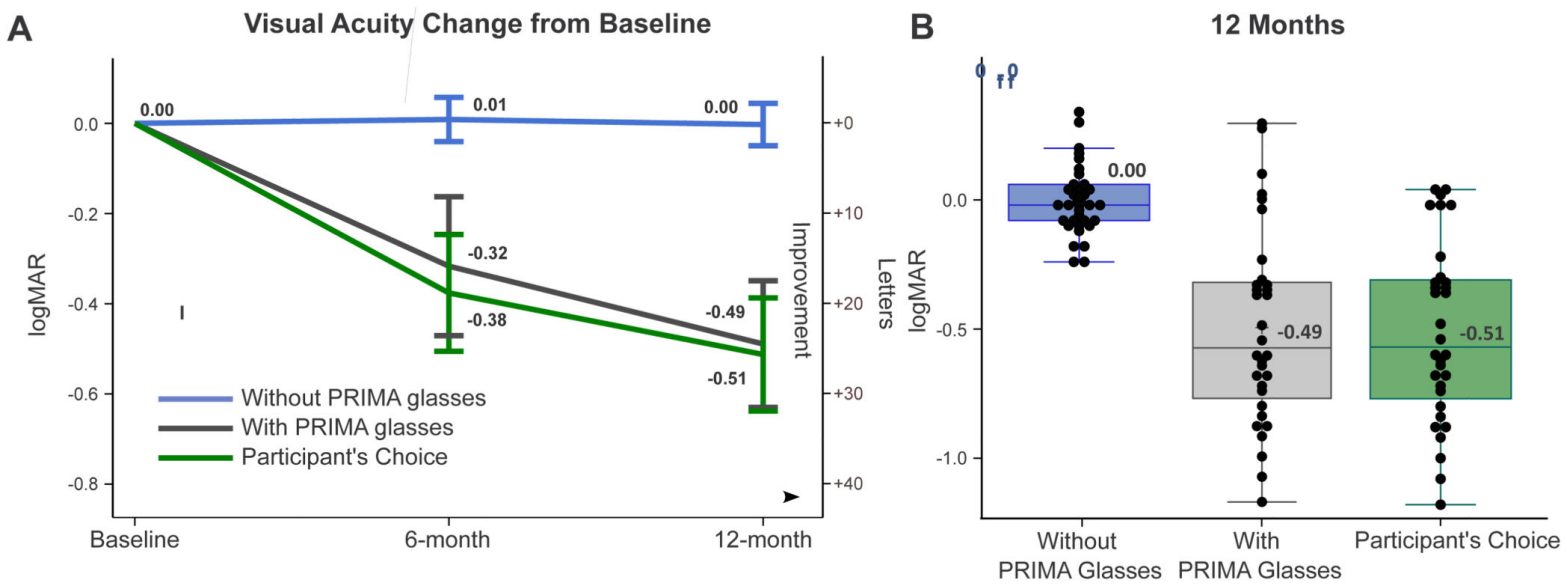
Change of VA from baseline (ETDRS). (A) Progression of VA over time, measured at baseline, 6 months, and 12 months under three conditions: without PRIMA glasses (blue line), with PRIMA glasses (grey line), and participant’s choice (green line). Change in VA is expressed in both logMAR (primary y-axis) and letters (secondary y-axis). Data points represent the mean, with error bars indicating the 95% confidence intervals. Negative logMAR values indicate improvement in VA. (B) Box plots depicting logMAR change from baseline at 12 months without PRIMA glasses, with PRIMA glasses and participant’s choice. Individual participants’ data are represented by black dots.

At 12 months, participants showed mean VA improvements under both conditions: with PRIMA glasses (logMAR 0.49; 95% CI: 0.35 to 0.63; range: -0.32 to 1.18) and with participant’s choice (logMAR 0.51; 95% CI: 0.39 to 0.64; range: -0.04 to 1.18), corresponding to improvements of 24.5 and 25.5 letters, respectively. With participant’s choice, those who used natural vision (N=4) had no meaningful VA improvement (logMAR -0.01; range: -0.04 to 0.02). The VA improvement, with predefined thresholds of logMAR 0 and 0.2, was found to be statistically significant using a sequential t-test (logMAR 0: p<0.001; logMAR 0.2: p<0.001) (Table S9). The best improvement was logMAR 1.18 (59 letters; Figure 3). At 6 months, 20 out of 35 participants had an improvement of logMAR 0.2. The mean improvement was higher from baseline with the PRIMA system (logMAR 0.32; 95% CI: 0.16 to 0.47) and with participant’s choice (logMAR 0.38; 95% CI: 0.25 to 0.51) (Figure 3). In contrast, when participants did not use PRIMA glasses, there was no change in VA from baseline (6 months: logMAR 0.01; 95% CI: -0.04 to 0.06; 12 months: logMAR 0.00; 95% CI: -0.05 to 0.04). Participants dynamically adjusted zoom settings (range 1×–12×) and brightness during VA testing (see Table S8 for maximum zoom usage). Single-participant VA trajectories and further information on VA outcomes based on years since AMD diagnosis prior to implantation can be found in Figure S2 and Tables S10 and S11.

Digital enhancement features including contrast, brightness, and zoom applied to the camera feed helped participants to see a wide range of visual stimuli. At 12 months, 84.4% of participants (27/32) could read letters, numbers and words using prosthetic vision at home (see supplementary videos S1–S3); 68% (22/32) reported medium-to-high user satisfaction with the PRIMA system. However, results from the IVI survey did not indicate behavioral changes (Table S12).

### Post-hoc analysis

After the primary analysis, a similar analysis was performed for an improvement of logMAR ≥0.3 and found 78.1% of participants demonstrated an improvement at 12 months (see Table 1).

### Safety

Twenty-six of device- and procedure-related SAEs occurred in 19 unique participants (Table 2) in 12 months. All SAEs were related either to the implantation procedure alone or a combination of the procedure and device. No SAE was related to the device alone. Of the 26 SAEs, 21 (81%) occurred within 2 months of surgery, of which 20 resolved within 2 months of occurrence. Twenty-two (85%) were classified as mild or moderate. Four (15%) were severe (macular hole, ocular hypertension, retinal detachment, proliferative vitreoretinopathy). The most frequent SAE was ocular hypertension (6 events; 23%), which occurred between 1 day and 3 weeks post-implantation, and all cases have been resolved. Five participants had peripheral retinal breaks; all were treated intraoperatively, and none resulted in rhegmatogenous retinal detachment. Three participants developed a subretinal hemorrhage during implantation, which stopped spontaneously, or after increasing infusion pressure. One had recurrent subretinal hemorrhage associated with choroidal neovascularization at 9 months. This participant and another, who also had choroidal neovascularization, were treated with intravitreal anti-vascular endothelial growth factor (VEGF) therapy. Three SAEs were full-thickness macular holes; two of these required moving the implant away from the hole to improve the functionality of the device. The choroidal fold, retinal detachment and proliferative vitreoretinopathy occurred in the same participant, but were successfully treated by surgery and silicone oil tamponade.

As demonstrated in Figure 2 and assessed by imaging (OCT, fundus photography), the inner retinal structure in the atrophic area does not appear to have been altered by the subretinal placement of the implanted chip.

The average area of atrophy in the study eyes increased from baseline to 12 months by 8.5 mm^2^, compared with 2.5 mm^2^ increase in the fellow eyes; this was attributed to post-surgical changes. There was no change in mean natural VA compared to baseline in the study eyes (Figure 3). Of the 32 patients at 12 months, 11 of the 14 participants affected by SAEs (79%) and 15 of the 18 participants unaffected by SAEs (83%) showed improvement of logMAR ≥0.2 with the PRIMA system at 12 months. All study-related SAEs were prespecified in the risk analysis, and none were life-threatening. On review of these and other data obtained in this study, the DSMB concluded that the PRIMA system’s benefits outweigh the risks of implantation.

## Discussion

The PRIMA system demonstrated meaningful visual improvement in participants with profound vision loss due to fovea-involving GA from AMD, in a representative population sample.^27^ This study demonstrated that 81% of participants at 12 months gained 10 or more letters (logMAR ≥0.2), and 78% of participants gained 15 or more letters (logMAR ≥0.3), with the PRIMA system. The mean improvement at 12 months was 25.5 letters (logMAR 0.51), and the maximum was 59 letters (logMAR 1.18). Moreover, 84% of participants reported using the device at home for reading letters, numbers, or words. They were able to read fonts smaller (up to 20/42 Snellen) than the sampling limit of 100 µm pixels (about 20/400 Snellen) using digital enhancements like zoom. Other low-vision aids like extraocular magnifiers or implantable telescopes enlarge images to enable utilization of the retina beyond the edges of atrophy^24,28^, achieves a typical average gain of logMAR 0.24^24^ and magnify the full visual field. In contrast, PRIMA restores vision to the area of scotoma and only magnifies the central prosthetic vision, leaving natural peripheral vision unaffected.^14^ While the native resolution of PRIMA approximates that of the peripheral retina in the participants (~logMAR 1.3), the additional abilities to zoom and enhance contrast and make other image improvements allowed participants to gain acuity beyond this level.

Prosthetic vision was previously attempted with the epiretinal implant Argus II,^16,17^ the subretinal implant Alpha IMS,^29^ and a 44 channel suprachoroidal implant.^30^ ARGUS II had limited resolution due to large electrode pitch (highest reported visual acuity was logMAR 1.8)^31^ and stimulation of axons from remote retinal ganglion cells, resulting in a distorted retinotopic map.^32,33^ Suprachoroidal implants are relatively far from the retinal neurons, and therefore provide comparatively low resolution in the range of logMAR 3.0.^30^

PRIMA allows the use of eye movement, as opposed to head-scanning,^23^ to reorient the camera. Eye-scanning improves resolution beyond the sampling limit,^34–36^ presumably for similar reasons as super-resolution algorithms implemented with conventional cameras.^37^ Unlike epiretinal implants, PRIMA stimulates bipolar cells rather than ganglia, which preserves features of inner retinal signal processing.^18,38^

Our data, collected over 12 months, demonstrate that PRIMA can be safely implanted under the atrophic macula, restoring central vision while preserving residual natural peripheral vision. Our previous feasibility study demonstrated stable anatomy with minimal reduced thickness of the inner retina over 36 months and VA logMAR 0.64 (32 letters) at 48 months.^21,39^ The wireless design simplifies implantation and reduces the surgical and post-operative risks compared to wired retinal implants with permanent openings of the eye globe.^31^ PRIMA is thin (about half the height of a photoreceptor) and integrates with the retina without mechanical fixation, such as retinal tacks used with other implants.^24^

The complications we observed in this study are consistent with the risks associated with vitrectomy and subretinal surgery, namely sub-retinal hemorrhage and CNV.^40,41^ Two participants (5.2%) developed CNV, which was successfully treated with intravitreal anti-VEGF therapy on a *pro re nata* regimen. CNV can occur in GA even without any intervention^5^ and has also been observed in patients following retinal surgical procedures. CNV incidence has been reported in vitrectomized eyes with dry AMD as 16% after 2.6 years.^42^

The implantation of a subretinal prosthesis may benefit from advanced intraoperative imaging technologies. Increased atrophy in the study eye frequently mirrored the areas of retinal bleb formation and retinotomy. Notably, no significant change in native vision was observed. The study was not powered to detect a difference in the IVI questionnaire and the questionnaire may be insufficiently sensitive for our participants, who have extremely low vision.

The 12-month results of this pivotal clinical trial demonstrated that the PRIMA subretinal implant can restore meaningful central vision in GA due to AMD, enabling visual tasks, such as reading and writing. While no implants have been explanted in humans, the wireless design allows for replacements with higher resolution next-generation chips,^43,44^ or multiple modules to “tile” the atrophic area with minimal incision.^45^

## Supplementary Material

Supplement

## Figures and Tables

**Table 1:** Primary efficacy endpoint: VA improvement of logMAR ≥0.2 and ≥0.3 at 12 months.

Group	Improvement logMAR ≥ 0.2		Improvement logMAR ≥ 0.3^c^		Participants
	n/N and % of Participants	95% CI p-value^‡^	n/N and % of Participants	95% CI	(Total; Imputed)
Primary Endpoint (Multiple Imputation)^a^	NA^†^/38 79.90%	65.58– 94.16% <.001	NA^†^/38 77.20%	62.18– 92.14%	38; 6
Observed Data^b^	**26/32** 81.30%	63.56– 92.79% <.001	**25/32** 78.10%	60.03– 90.72%	32; 0

Binomial test results of two different scenarios.

a Primary Endpoint (Multiple Imputation): Multiple Imputation analysis was performed on missing data.

b Observed Data: Analysis of observed data on implanted participants with a non-missing 12-month follow-up result.

c Post-hoc analysis.

† The multiple imputation results do not provide the exact proportion of participants achieving the primary efficacy endpoint but rather an estimated percentage, computed over 100 imputed data sets.

‡ The p value is given for the null hypothesis of proportion of participants - with an improvement of logMAR 0.2 or more - less than or equal to 50%.

Exact intervals were used when analyzing observed data while Logit intervals were used for multiple imputation.

**Table 2:** Device- or procedure-related SAEs at the 12-month follow-up.

SAE Type	N participants with SAE	% participants with SAE	Number of events
Ocular hypertension	6	15.79%	6
Peripheral retinal break	5	13.16%	5
Macular hole	3	7.89%	3
Subretinal hemorrhage	3	7.89%	4
Choroidal neovascularization	2	5.26%	2
Choroidal hemorrhage	1	2.63%	1
Choroidal fold	1	2.63%	1
Proliferative vitreoretinopathy	1	2.63%	1
Retinal detachment	1	2.63%	1
Retinal hemorrhage	1	2.63%	1
Thrombophlebitis	1	2.63%	1
**Total**	**25**		**26**
