# Supplementary material for: Vision Restoration with the PRIMA System in Geographic Atrophy Due to AMD
Source: N Engl J Med. Author manuscript; Available in PMC 2026 Jan 15. (PMC7618305; doi:10.1056/NEJMoa2501396)
Supplement: Supplement [file EMS207931-supplement-Supplement.pdf]

Vision Restoration with the PRIMA System in Geographic Atrophy Due to AMD

Supplementary Appendix

Authors:

Frank G. Holz, M.D., Yannick Le Mer, M.D., Mahiul M.K Muqit, M.D. PhD, Lars-Olof Hattenbach, M.D. PhD, Andrea Cusumano, M.D. PhD, Salvatore Grisanti, M.D., Laurent Kodjikian, M.D. PhD, Marco Andrea Pileri M.D., Frederic Matonti, M.D. PhD, Eric Souied, M.D. PhD, Boris V. Stanzel, M.D. PhD, Peter Szurman, M.D. PhD, Michel Weber, M.D. PhD, Karl Ulrich Bartz-Schmidt, M.D., Nicole Eter, M.D., Marie Noelle Delyfer, M.D. PhD, Jean François Girmens, M.D., Koen A. van Overdam, M.D. PhD, Armin Wolf, M.D. PhD, Ralf Hornig, PhD, Martina Corazzol, PhD, Frank Brodie, M.D., Lisa Olmos De Koo, M.D. MBA, Daniel Palanker, PhD, José-Alain Sahel, M.D.

Table of Contents

Participant criteria 4

Pre-implantation planning 7

Implantation procedures 7

Pre- and post-implantation examinations 8

Visual acuity measurements 8

Participant’s Choice 9

Measurements of prosthetic visual acuity without zoom: the implant resolution test 9

Theoretical resolution of the implant 9

Multiple imputation 10

Home use questionnaire 11

Tuning and training sessions 11

Central perception test 11

IVI (Impact of Vision Impairment) questionnaire 11

Correlation analysis of retinal thickness data 13

Figures 15
Figure S1. CONSORT flow diagram illustrating the progression of the PRIMAvera clinical trial 15
Figure S2. Single participant visual acuity (in logMAR) values at baseline and 12 months, comparison between with PRIMA Glasses and Participant's Choice 16
Tables 17
Table S1. Examinations performed only pre-implantation for participant inclusion 17
Table S2. Ophthalmological examinations and refraction measurement (pre and post implantation) 17
Table S3. Imaging and microperimetry (pre and post implantation) 18
Table S4. Demographics and characteristics of participants at baseline 21
Table S5: Overall representativeness of the AMD and GA population 22
Table S6. Participants not assessed at 12-month follow-up 22
Table S7. Implant resolution test at 12 months 23
Table S8. Maximum zoom used for reading of penultimate line in ETDRS chart 23
Table S9. Secondary efficacy: Visual Acuity improvement at 12 months, Participant's Choice 24
Table S10. Visual acuity (mean ± SD) at baseline and 12-month follow-up: comparison of observed data vs. all participants 24
Table S11. Visual acuity outcome at 12 months based on years since AMD diagnosis prior to implantation 25
Table S12. Comparison of IVI percentile scores in participants at baseline, 6 and 12 months 25

Table S13. Correlation analysis of retinal thickness at baseline vs. visual acuity with PRIMA glasses at 12 months (in logMAR) ... 26

Videos ... 26

Video S1. Reading text ... 26

Video S2. Writing ... 26

Video S3. Playing cards ... 26

References ... 27

Participant criteria

Inclusion Criteria

- Is 60 years or older at the date of inclusion;
- Has a confirmed diagnosis of geographic atrophy due to AMD in both eyes;
- The study eye has best corrected visual acuity of logMAR 1.2 (20/320) or worse as measured by ETDRS test;
- Has an atrophic patch in the study eye including the fovea of at least the implant size (>4.5mm^2 and >2.4 mm in minimum diameter);
- Understands the constraints of the study and accepts to present for all scheduled follow up visits;
- Participant signed informed consent.

Exclusion criteria

A participant was excluded from the study if he or she:

- Had cataract in the study eye (with LOCS III scale NO, NC, C or P>1); (these participants were asked to have cataract surgery performed prior to enrollment; all other participants got IOL replacement during the PRIMA implantation);
- Underwent intra ocular lens implantation in the study eye within the last month;
- Had a highly myopic study eye (>26 mm axial length [AL]);
- Had a highly hyperopic study eye (<20 mm AL);
- Had no light perception in either eye;
- Had a history of documented choroidal neovascularization in either eye;
- Had any signs of exudative AMD including exudative AMD with detachment of retinal pigment epithelium in the central visual field of the study eye;
- Had an implanted telescope in one eye;
- Had a black IOL in the study eye;

- Had any disease (other than study allowed diseases) or condition that affects retinal function of the study eye or the visual system (e.g., central retinal artery/vein occlusion, end-stage diabetic retinopathy, Proliferative Diabetic Retinopathy (PDR), diabetic macular edema (DME), severe Non-Proliferative Diabetic Retinopathy (NPDR), retinal detachment, infectious or inflammatory retinal disease, severe glaucoma, optic neuropathy, etc.);
- Had any disease or condition that prevented adequate examination (including OCT) of the study eye including but not limited to media opacities that cannot be resolved prior to implantation. Note that this criterion is also important for the function of the implant.
- Had a corneal endothelial cell count of less than 1000 cells/mm² in the study eye;
- Suffered from nystagmus or other ocular motility disorders;
- Had any disease or condition that precludes the understanding or communication of the informed consent, study requirements or test protocols (e.g., deafness, severe multiple sclerosis, amyotrophic lateral sclerosis, severe neuritis, etc.);
- Had epileptic seizures;
- Had a known sensitivity to the contact materials of the implant (iridium oxide, silicon-carbide, and titanium);
- Had a known allergy to anesthetic drugs;
- Presented with hypotony in the study eye (<8 mmHg);
- Presented with hypertension in the study eye (>23 mmHg with treatment);
- Had active cancer or a history of intraocular, optic nerve or brain cancer and metastasis;
- Was an immune-suppressed participant (e.g., due to HIV positive diagnosis etc.);
- Was a known carrier of multi-resistant microorganisms;
- Was receiving anticoagulation therapy that cannot be adapted to allow eye surgery;

- Was participating in another investigational drug or device study that may interfere with the PRIMAvera study;
- Had a history of chronic or recurrent infection or inflammation that would preclude participation in the study;
- Had significant recurrent or chronic inflammations or infections. Specifically, participants with the following disorders are excluded:
    - Severe chronic and consuming diseases that frequently associated with infection (e.g. Crohn's disease, Whipple's disease);
    - Active inflammation in the area of the eye (e.g. herpes of cornea and/or conjunctiva, recurrent blepharoconjunctivitis, hordeolum, chalazion);
- Had a severe psychological disorder;
- Did not have the mental capacity to legally sign the informed consent;
- Had severe renal, cardiac, hepatic, etc. organ diseases (ASA IV or worse);
- Had head dimensions that are incompatible with the PRIMA glasses;
- Had a refraction of study eye higher than + 4 dpt or lower than – 4 dpt for participants with IOL (there is no refraction criteria for phakic participants, since they received an IOL during PRIMA implantation);
- Had too high and/or unrealistic expectations (e.g., believes that a benefit is guaranteed or expects normal vision after surgery).

The following additional exclusion criteria were applicable for French participants:

- Was a protected person per French law (e.g. is under guardianship, person deprived of their liberty);
- Was not affiliated to a mandatory social security program (health insurance)

Pre-implantation planning

The eye with the worst VA was selected as the study (implanted) eye. In cases where both eyes had similar VA (max difference logMAR 0.2), the participant and surgeon chose the study eye. Microperimetry data was mapped onto FAF to determine the participant's preferred locus for eccentric fixation. This area, along with retinal vessels were avoided in planning of the retinotomy for device introduction.

Implantation procedures

Surgical procedure and vision training

The subretinal photovoltaic array was implanted in conjunction with a pars plana vitrectomy. The retina was detached outside the area of GA in the pre-specified location by injecting balanced salt solution (BSS) with a 41-gauge cannula. Following application of diathermy for hemostasis of potential vessels, a 3mm retinotomy was created in the detached retina. Areas of residual retina attachment within the GA where the implant was to be placed were gently elevated using a spatula. Once a sufficient area of GA was elevated, a limited peritomy and 3mm sclerotomy were performed, aligned with the retinotomy and the target implant position. The PRIMA Delivery System loaded with an implant was introduced through the sclerotomy and aligned with the retinotomy. The implant was injected into the subretinal space and gently positioned. Heavy perfluorocarbon liquid was then placed on the macula to induce egress of residual subretinal fluid and flatten the retina on top of the implant. The sclerotomy was then sutured and no laser photocoagulation of the retinotomy was required. The surgeon verified the implant's correct orientation with the electrodes side facing up. If necessary, the implant was moved closer to the fovea using a spatula or Thomas pick transretinally. A fluid/air exchange was performed to remove the perfluorocarbon liquid and BSS. Endotamponade was injected per surgeon's preference (either gas or silicone oil). The sclerotomies were closed and the conjunctiva was sutured. Participants were kept in a supine

position for at least one-hour post-procedure, to prevent implant migration. Post implantation, participants were treated according to the hospital standard of care for intraocular surgeries.

Pre- and post-implantation examinations

Participants underwent a series of examinations pre-implantation (Table S1) to confirm the diagnosis prior to inclusion including measurement of atrophy size. Pre- and post-implantation participants underwent complete ophthalmic evaluation with refraction (Table S2) fundus photography, FAF, optical coherence tomography (OCT) and fluorescein and indocyanine angiography as well as microperimetry (Table S3). In follow up examinations these were used to monitor safety and assess accuracy of the implant location within the area of GA. All imaging data were assessed by a third-party reading center, Steinbeis Transfer GmbH (GRADE Reading center). Grading of images was performed at 4 weeks post-op to confirm the implantation compliance (defined as the placement of the PRIMA implant in the subretinal space with the center of the implant within the atrophy- associated scotomata) and at 12 months. Four to five weeks following implantation surgery, participants began vision training to learn how to use the system.

Ophthalmological assessments were conducted at defined time intervals after implantation to confirm healing, assess the outcomes, and observe any complications (Table S2). Vision was also assessed using questionnaires at baseline, 6 months, and 12 months.

Visual acuity measurements

Visual acuity was measured with standard ETDRS charts at 1 meter distance. Participants were asked to start at the top of the chart and read as many letters as possible. Measurements were done at baseline without the PRIMA glasses and at the 6-month as well as at 12-month follow up with the PRIMA glasses and without. To improve the accuracy for the primary endpoint measurement on the study eye, the tests were done 3 times at baseline (without the PRIMA glasses) and at 12 months 3 times with the PRIMA glasses and 3 times without.

The median value of these three measurements was then used for analysis of each condition. To avoid fatigue effects, all measurements on the study eye were done on different days. When VA was measured without the PRIMA glasses best corrective lenses were used.

Participant's Choice

At each follow-up, the participant's visual acuity was measured with and without the PRIMA glasses. Analysis was then performed under three conditions: 1) without PRIMA glasses, 2) with PRIMA glasses, and 3) participant's choice (the better visual acuity out of the first two conditions) to reflect home use, based on the assumption that, when the PRIMA glasses provided no benefit, participants relied on their natural visual acuity. The participant's choice condition was introduced because the PRIMA glasses partially obscured the residual visual field of the participant. As a result, when the PRIMA glasses did not improve VA, the measured VA with PRIMA glasses might have underestimated the participant's real acuity. The participant's choice condition thus mitigated this potential bias.

Measurements of prosthetic visual acuity without zoom: the implant resolution test

The purpose of this test was to measure visual acuity that a PRIMA implant could provide independently from external factors, such as head movement, image processing of the PRIMA system, or zoom. Landolt ring patterns were presented on the display of the PRIMA glasses directly, i.e. without the use of the camera. The Landolt ring has one of four orientations (opening up, down, right, or left) and the participant was asked to tell the orientation. The threshold where the participant detected the orientation was determined by a 1up/2down staircase method.

Theoretical resolution of the implant

Visual acuity achievable with the PRIMA implant was constrained by its pixel size. In the context of normal human vision (logMAR 0.0 or Snellen 20/20), the corresponding angular

frequency is 30 cycles per degree. Given that one degree on the human retina spans approximately 288 μm, a single cycle corresponds to 9.6 μm (288 μm/30). Resolving one cycle requires at least two pixels: one for the black band and one for the white band of the grating, resulting in an equivalent pixel size of 4.8 μm for normal vision. With a pixel size of 100 μm, as in the PRIMA implant, the resolution is about 21 times lower than normal, corresponding to a visual acuity of logMAR 1.32 (Snellen 20/417). Multiple samplings of the image due to eye movements help improve resolution beyond the sampling limit by as much as 30%, presumably based on similar principles to the super-resolution algorithms implemented with conventional cameras. Additional sharpening of the image may be a benefit of two diodes per pixel in the PRIMA implant, where both diodes should be illuminated to generate electric current.

Multiple imputation

Multiple imputation analysis of the primary efficacy results was used to prevent bias due to exclusion of participants with missing data, enabling analysis of the full (N=38) set of participants. Pre-specified covariates were used in the model to avoid selection bias and reduce the risk of type I error inflation. These covariates were 6-month implant resolution test score, 6-month visual acuity with PRIMA glasses, 6-month home-use questionnaire, age, and sex. Since no participants withdrew from the study prior to 12 months due to insufficient benefit from the PRIMA system, all missing data for the primary efficacy endpoint were addressed using multiple imputation. Multiple imputation was based on fully conditional specification using both linear and logistic regression. A total of 100 imputed datasets were generated and combined using Rubin's rules. The estimated proportion of successes and the associated asymptotic standard error were combined to produce the final estimated success proportion and its associated confidence interval.

Home use questionnaire

As part of the evaluation, the PRIMA system was tested in participants' home environments following initial rehabilitation training to assess its integration into daily life and its impact on routine task performance. The questionnaire included 19 questions regarding the system's use at home, with possible answers being either Yes or No. For inclusion in the multiple imputation model, the total score from the 19 items was used.

Tuning and training sessions

Participants received up to 40 training sessions within the first 12 months. Training sessions focused on adapting to the PRIMA glasses and to prosthetic vision, starting with simple tasks, such as detecting objects on high contrast background, and progressing to more challenging goals, such as reading letters, numbers, and words in a variety of settings.

Central perception test

At baseline, participants had little to no perception (0.5 ± 1.5dB) at the future implant location, as measured by microperimetry. At 12 months, various sequences of artificial stimuli were projected with the PRIMA system, equivalent in intensity to the stimulus projected by the microperimeter (24dB). Thirty participants demonstrated central perception. Among the thirty participants with central perception, twenty-four showed reliability against false positives. Improvement was defined as the difference between the sensitivity threshold measured with the PRIMA system (24 dB) and the baseline sensitivity. The improvement in sensitivity measured across participants was 23.37dB.

IVI (Impact of Vision Impairment) questionnaire

The study utilized a 28-item questionnaire concerning various aspects of daily life administered at baseline, 6 and 12 months. The questionnaire was divided into three subscales: reading and accessing information, mobility, and independence, as well as emotional well-being. Items 1-

13 were rated on a 4-point Likert-type scale ranging from "Not at all" to "A lot" plus a non-applicable option "Does not apply to me." Items 14-15 were rated on a 3-point Likert-type scale ranging from "Not at all" to "A lot" plus a non-applicable option "Does not apply to me." Items 16-28 were rated on a 4-point Likert-type scale ranging from "Not at all" to "A lot of the time." Higher scores indicated better quality of life outcomes. The "Does not apply to me" response was not included in computing the average overall or domain score. Before the implantation, each question was asked once. After implantation, each question was asked twice: first, without the PRIMA system, and second, with the option to use the PRIMA system or not (participant's choice). For each question a score was given.

Rasch-transformed person measures for the overall IVI and three subscale scores were used for statistical analyses. Mean IVI scores rated by participants when using and not using the PRIMA system were compared using a paired t-test. Changes in mean IVI scores from baseline over time (6, 12 months) were estimated using linear repeated measure regression models, with fixed effect parameters for time (treated as a categorical variable) and a compound symmetric covariance structure to account for within-participants correlation. Least-square mean estimates at each time point and the associated 95% confidence intervals were provided. Analyses were performed separately for the overall score and for the three subscales.

The IVI results suggest low response to the overall IVI and the three subscales when evaluating PRIMA intervention. However, this diverges from clinical observations. Notably, an additional qualitative measure – the home use questionnaire - collected during the trial indicated improvements in quality of life and successful home use of the system. The home use questionnaire is a non-validated tool developed to understand how the PRIMA system is used at home.

Several potential factors may explain the low response of the overall and subscales IVI scores to the PRIMA intervention. Not all IVI subscales, and consequently the overall IVI, align with the key functional domains of the PRIMA system. For example, mobility and independence are not primary objectives during the first 12 months of PRIMA use. This can potentially introduce and spread noise in the measurements. Additionally, some IVI single items may not have been appropriately calibrated to the participants' level. While the IVI has some specific reading items, these may not have been at the correct level for participants. For example, reading newspaper print (ordinary print size), may have been too difficult for most of the participants both with and without PRIMA. In fact, what is usually achieved with the PRIMA system was reading headlines or bigger text. While the PRIMA system showed significant improvement of visual acuity, the reading tasks of the IVI questionnaire requires greater acuity than achieved with the current generation device. None of the reading items appear to be fully aligned with the achievements made possible by the PRIMA system. The miscalibration of these reading questions poses a significant challenge in evaluating the PRIMA intervention on the IVI questionnaire, as reading and accessing information was the most relevant subscale where a potential effect could have been observed.

Correlation analysis of retinal thickness data

To evaluate the correlation between retinal thickness and visual acuity with PRIMA glasses, central subfield retinal thickness (CSRT) data at baseline was analyzed and compared to the 12-months visual acuity outcome. Central subfield retinal thickness (CST) was defined by the average of all points within the inner circular area of 1 mm radius centered on the umbo.

Due to the non-normal distribution of the retinal thickness data (mean ± SD:157 ± 41,84 µm), Spearman correlation coefficients were computed. Correlations were computed for absolute visual acuity with PRIMA glasses as well as visual acuity gain at 12 months. As shown in Table

S13, retinal thickness data at baseline showed no correlation with visual acuity (Spearman's ρ = 0.16, p=0.384) or visual acuity improvement (Spearman's ρ = 0.153, p=0.402) at 12 months.

Figures

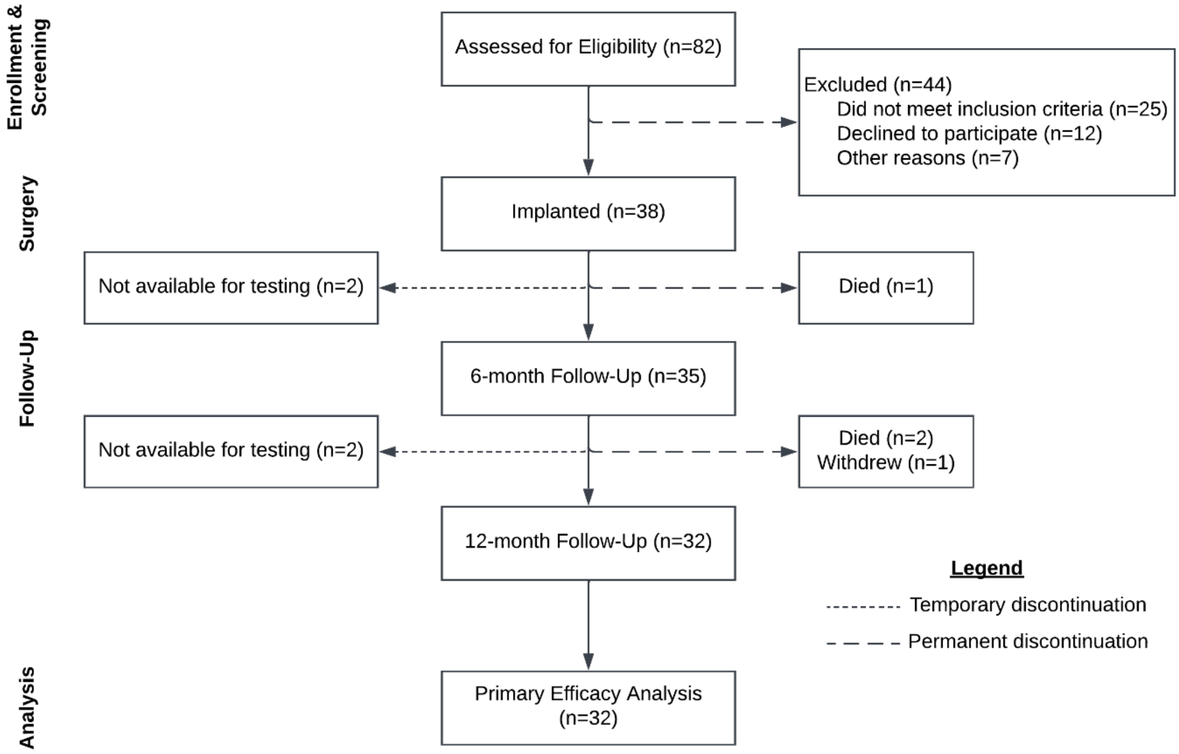

Figure S1. CONSORT flow diagram illustrating the progression of the PRIMAvera clinical trial. A total of 82 participants were assessed for eligibility, of whom 38 were implanted. Follow-up VA data was collected at 6 and 12 months. Attrition occurred due to temporarily discontinued interventions (depicted by the left dotted arrows) and permanent loss to follow-up or deaths (depicted by the right dashed arrows). 32 participants were included in the primary efficacy analysis.

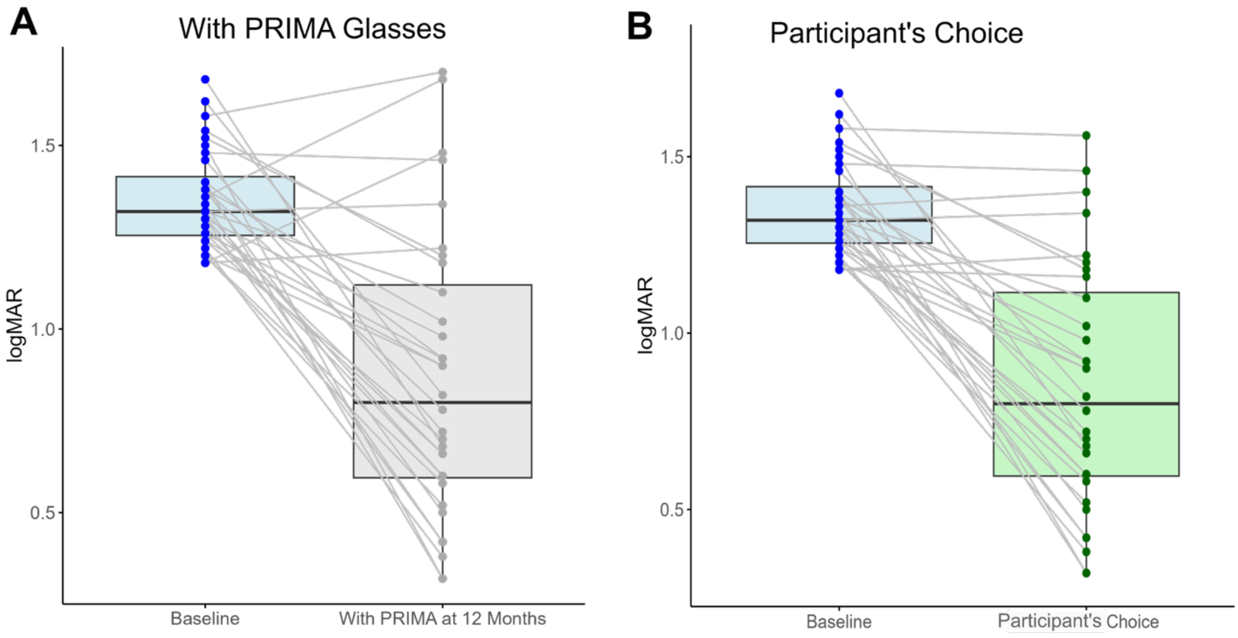

Figure S2. Single participant visual acuity (in logMAR) values at baseline and 12 months, comparison between with PRIMA Glasses and Participant's Choice. Boxplots of visual acuity data distribution showing single participant's values for baseline, in blue, with PRIMA glasses at 12 months, in gray, and for Participant's Choice at 12 months, in green.

Tables

Table S1. Examinations performed only pre-implantation for participant inclusion

Examination	Description
Medical history	Information related to the participant's past medical history, medications, demographic information, and disease state was recorded
Endothelial cell count	Measurement of endothelial cells of the cornea
Orthoptic assessment	Evaluation of the binocular vision of a participant to reveal any condition that could strongly impact the participant's possibility of using the PRIMA system after implantation.
Mental State Exam questionnaire	This questionnaire provides support in assessing the cognitive aspects of mental function of participants at baseline

Table S2. Ophthalmological examinations and refraction measurement (pre and post implantation)

Examination	Description
Tonometry	Intra ocular eye pressure was measured
Slit-lamp	The participant's anterior segment of the eye was examined with a slit-lamp
Fundoscopy	The posterior part of the eye is examined with a slit lamp and indirect ophthalmoscopy. The vitreous, macular and the peripheral retina is are each assessed
Refraction measurement	Obtained manifest refraction to customize the lenses of the PRIMA Glasses and to identify the best correction for the visual acuity tests.<br>Manifest refraction was measured with the Snellen Eye Chart.

Table S3. Imaging and microperimetry (pre and post implantation)

**Examinations**	
**Color fundus photography (FP)**	
**Description**	A color image of the retina was acquired
**Tools**	● Standard digital fundus camera<br>● Angle of view: 45° - 60°, or Zeiss Clarus 500/700<br>● Minimum resolution for images: 1536 x 1536 pixels
**Protocol**	Study eye<br>● Color Fundus reflex (Mono) n = 1<br>● Color Fields 1M, 2, 3M* (Mono) n = 3<br>● Red free Field 2* (Mono) n = 1<br>Fellow eye<br>● Fundus reflex (Mono) n = 1<br>● Fields 1M, 2, 3M* (Mono) n = 3<br>● Red free Field 2* (Mono) 1 Field 2* (Mono) n = 1<br>Field 1M (Disc): Temporal edge of the disc in the center of the image<br>Field 2 (Macula): Fovea in the center, temporal part of the disc at the edge of the image<br>Field 3M: Temporal part of the macula together with the center of the fovea with approximately one disc diameter to the edge of the image
**Indocyanine angiography (ICG)**	
**Description**	ICG angiography images were acquired

<table>
<tr><td>Tools</td><td>Use of the confocal scanning laser ophthalmoscopy, i.e., the Spectralis HRA+OCT, FA+OCT, or the Spectralis HRA was performed for FA and ICGA. Alternatively, the same fundus camera as for fundus photography (for either one or both FA+ICGA) was utilized if it fulfilled the following minimal requirements:
● Digital
● Angle of view: 45° - 60°, or Zeiss Clarus 700
● Minimum resolution for black & white shots: 1536 x 1536 pixels</td></tr>
<tr><td colspan="2">Fluorescein Angiography (FA)</td></tr>
<tr><td>Description</td><td>Fluorescein angiography images were acquired</td></tr>
<tr><td>Tools</td><td>See ICG</td></tr>
<tr><td>Protocol</td><td>See ICG</td></tr>
<tr><td colspan="2">Autofluorescence imaging</td></tr>
<tr><td>Description</td><td>Autofluorescence images were acquired</td></tr>
<tr><td>Tools</td><td>The Spectralis models (manufacturer Heidelberg Engineering GmbH/Inc.) listed below with Spectralis Software Version 6.12a or newer must be used:
● Spectralis HRA+OCT (for SD-OCT, FAF)
● Spectralis OCT with Bluepeak module (for SD-OCT, FAF)
● Spectralis HRA and HRA2 (for FAF)</td></tr>
<tr><td>Protocol</td><td>● One field 2 (30° x 30°) near-infrared (NIR) reflectance image.
● One field 2 (30° x 30°) blue reflectance (so called “red-free”) image.
● One field 2 (30° x 30°) FAF image
● One field 2 (55°) near-infrared (NIR) reflectance image.
● One field 2 (55°) FAF image</td></tr>
<tr><td colspan="2">Optical Coherence Tomography (OCT)</td></tr>
</table>

**Description**	OCT volume scans were acquired with a SPECTRALIS (Heidelberg Engineering GmbH/Inc, Germany.)
**Tools**	The Spectralis models (manufacturer Heidelberg Engineering GmbH/Inc.) listed below with Spectralis Software Version 6.12a or newer were used:<br>● Spectralis HRA+OCT (for SD-OCT, FAF)<br>● Spectralis OCT with Bluepeak module (for SD-OCT, FAF)<br>● Spectralis HRA and HRA2 (for FAF)
**Protocol**	● SD-OCT Protocol 1 –Seven-line horizontal raster-scan<br>● SD-OCT Protocol 2 – Seven-line vertical raster-scan<br>● SD-OCT Protocol 3 – horizontal dense volume scan (30° x 30° lens), centered on fovea<br>● SD-OCT Protocol 4 – vertical dense volume scan (30° x 30° lens), centered on fovea<br>● Protocol 5 – volume scan (30° x 30° lens), centered on implant and scan axis aligned with edge of the implant on one side<br>● Protocol 6 – volume scan (30° x 30° lens), centered on implant and scan axis aligned with edge of the implant on the other site
**Microperimetry**	
**Description**	Microperimetry (MAIA, MP-1 or MP-3) measures retinal sensitivity thresholds and the preferred retinal location (PRL)
**Tools**	● CenterVue MAIA (preferred system)<br>● NIDEK MP-3<br>● NIDEK MP-1
**Protocol**	Condition: Mesopic (background luminance = 4 asb)<br>Test Grid: Built-in grid 10-2, consisting of 68 stimuli<br>Stimulus color: White

Table S4. Demographics and characteristics of participants at baseline

Characteristic	All Treated (N=38)
Age at Inclusion (years)	
Mean ± SD	78.9 ± 6.41
Range	67 - 92
Median (Q1, Q3)	78 (75, 83)
Gender, N (%)	
Female	20 (52.6)
Male	18 (47.4)
Implanted eye, N (%)	
Left	20 (52.6)
Right	18 (47.4)
Atrophic area	
Mean ± SD	24.7 ± 12.33
Years since AMD diagnosis at time of implant (N=36)	
Mean ± SD	9.7 ± 6.07
Microperimetry retinal sensitivity in the implant area (dB)	
Mean ± SD	0.5 ± 1.5

Table S5: Overall representativeness of the AMD and GA population

Age	Prevalence of AMD and GA increases with age [1,2]
Sex and Gender	Geographic Atrophy is more common in women [3]
Race and Ethnicity	Geographic atrophy is more common in Caucasians [4]
Geography	More common in Europeans and those with European ancestry [3.4]
Overall representativeness of the trial	Increasing age is the largest risk factor for GA associated with AMD [1,4] and our participant's mean age of 78.9 years (±6.41 SD) fits within the representative age range for advanced AMD.
	Our study showed a slight preponderance of female participants versus male participants, in keeping with the fact that GA associated with AMD is more common in women.[3]
	Most of our study centers were in Europe which has the most common documented geographic incidence of AMD.[3,4]

Table S6. Participants not assessed at 12-month follow-up

Reason for missing Primary Endpoint	Number of participants	Comment
Death	3	• Death following fall (non-study related)<br>• Intracerebral mass hemorrhage due to hemorrhagic diathesis (non-study related)<br>• Natural death (non-study related)
Not available for testing	2	• Broken left wrist prevented participant from attending the 12-month visit (non-study related AE)<br>• Presence of silicone oil in study eye*
Withdrawal	1	• Participant withdrew consent due to availability, change of domicile, health issue

*Approximately six months after implantation, the participant developed proliferative vitreoretinopathy, which was successfully treated with retinotomy and silicone oil tamponade. The surgeon decided to keep the silicone oil in the eye to prevent further complications. While silicone oil in principle does not prevent the PRIMA implant from working, further regulatory approval was deemed to be necessary before the implant could be activated in the presence of silicone oil.

Table S7. Implant resolution test at 12 months

Visit	Statistics	Raw Score (logMAR) N=33[§]
12 months	Mean ± SD	1.32 ± 0.16
	Median (Min, Max)	1.33 (1.05, 1.60)
	Q1, Q3	1.23, 1.40

§ N = 33 The site started with Implant Resolution test, then the participant was not available for visual acuity with PRIMA system test due to a non-related adverse event.

Table S8. Maximum zoom used for reading of penultimate line in ETDRS chart

Visit	Zoom Used	Number (%) of Participants
12 months	1: 2	2 / 31 (6.45%)
	1: 4	6 / 31 (19.35%)
	1: 6	4 / 31 (12.90%)
	1: 8	17 / 31 (54.84%)
	1:10	1 / 31 (3.23%)
	1:12	1 / 31 (3.23%)

Table S9. Secondary efficacy: Visual Acuity improvement at 12 months, Participant's Choice

Group	Parameter	Value
Participant's Choice	Mean improvement and standard deviation of Visual Acuity (logMAR) at 12 months	0.51 ± 0.348
	N	32
	Two-Sided 95% Confidence Interval	0.387, 0.638
	p-value [a]	<.001
	p-value [b]	<.001

Only participants with non-missing baseline visit and 12 month visit data were included within this analysis.
P-value from paired t-test.
[a] Based on hypothesis test of zero improvement.
[b]Based on hypothesis test a logMAR Improvement of 0.2.

Table S10. Visual acuity (mean ± SD) at baseline and 12-month follow-up: comparison of observed data vs. all participants.

		Without PRIMA			With PRIMA
**Visit**	logomark	All implanted (n = 38)	Observed *without* PRIMA at 12 Months (n = 34)	Observed *with* PRIMA at 12 Months (n = 32)	Observed *with* PRIMA at 12 Months (n = 32)
**Baseline**	Mean ± SD	1.36 ± 0.139	1.36 ± 0.141	1.35 ± 0.132	NA
**12 Months**	Mean ± SD	NA	1.37 ± 0.166	1.34 ± 0.148	0.87 ± 0.387

n = Number of Participants

Table S11. Visual acuity outcome at 12 months based on years since AMD diagnosis prior to implantation.

	≤ 7.5 years	> 7.5 years	p-value
n/N (Total)	13/18 (32)	13/14 (32)	0.304
% ≥ logMAR 0.2	72.20%	92.90%	
95% CI	46.52%, 90.31%	66.13%, 99.82%	
N	18	14	0.177
Mean Change in VA	-0.41 ± 0.43	-0.6 ± 0.31	
95% CI	-0.62, -0.19	-0.78, -0.42	

Table S12. Comparison of IVI percentile scores in participants at baseline, 6 and 12 months

Participant's Choice (mean [95% Confidence Interval])‡				
	Baseline (N = 38)	6 months (N = 35)	12 months (N = 32)	Overall p-value*
Emotional [a]	-0.64 (-1.83, 0.54)	-0.47 (-1.99, 1.04)	0.41 (-1.03, 1.86)	0.12
Mobility [b]	-0.62 (-1.42, 0.17)	-0.81 (-1.87, 0.24)	-0.43 (-1.39, 0.51)	0.70
Reading [c]	-2.09 (-2.87, -1.30)	-1.75 (-2.65, -0.84)	-1.47(-2.15, -0.79)	0.30
Overall [d]	-0.88 (-1.55, -0.22)	-0.83 (-1.56, -0.12)	-0.45 (-1.09, 0.16)	0.35
Without PRIMA (mean[95% Confidence Interval])‡				
	Baseline (N = 38)	6 months (N = 36)	12 months (N = 34)	Overall p-value*
Emotional [a]	-0.57 (-1.70, 0.56)	-0.46 (-1.77, 0.83)	0.22 (-1.16, 1.61)	0.22
Mobility [b]	-0.62 (-1.42, 0.17)	-0.88 (-1.88, 0.11)	-0.53 (-1.49, 0.43)	0.68
Reading [c]	-2.09 (-2.87, -1.30)	-1.79 (-2.66, -0.91)	-1.61 (-2.28, -0.93)	0.48
Overall [d]	-0.88 (-1.55, -0.22)	-0.90 (-1.55, -0.25)	-0.58 (-1.22, 0.05)	0.43

*Estimated using repeated measures ANCOVA, adjusted for study site
†No significance found between any of the measures in pairwise comparison.
‡Adjusted means estimated using repeated measures ANCOVA
Degree of freedom = 2 for all analyses
Emotional without PRIMA: total sum square = 8.54; Emotional Participant's Choice : total sum square = 13.80
Mobility without PRIMA: total sum square = 1.61; Mobility Participant's Choice : total sum square = 0.81
Reading without PRIMA: total sum square = 2.66; Reading Participant's Choice : total sum square = 3.79
IVI without PRIMA: total sum square = 1.50; IVI Participant's Choice : total sum square = 2.27

[a]Scores ranged between -5.51 and 5.72 in the Without PRIMA group and between -5.51 and 5.72 in Participant's Choice group
[b]Scores ranged between -6.2 and 6.33 in the Without PRIMA group and between -6.2 and 6.33 in Participant's Choice group
[c]Scores ranged between -5.42 and 2.19 in the Without PRIMA group and between-5.42 and 2.19 in Participant's Choice group
[d]Scores ranged between -4.68 and 2.53 in the Without PRIMA group and between -4.68 and 2.8 in Participant's Choice group

Table S13. Correlation analysis of retinal thickness at baseline vs. visual acuity with PRIMA glasses at 12 months (in logMAR)

	**Spearman's ρ**	**p-value**
Visual acuity at 12 months (N = 32)	0.159	0.384
Improvement in visual acuity at 12 months (N = 32)	0.153	0.402

Videos

Video S1. Reading text. The first video presents a participant using the PRIMA system while reading text on paper positioned ~40cm away. The participant demonstrates the ability to read fluently, with a steady reading pace.

Video S2. Writing. The second video showcases a participant solving a crossword puzzle. The participant successfully completed the puzzle which required several key visual skills, such as identifying the correct space for writing, precisely locating the tip of the pen, and accurately writing the appropriate letter using hand-eye coordination skills.

Video S3. Playing cards. The third video features a participant playing cards while using the PRIMA system. The participant successfully located the card symbol and read it aloud. This demonstrates the participant's ability to visually identify and interpret card details accurately in real-time.

References

1. Rahimy E, Khan MA, Ho AC. Progression of geographic atrophy. Ophthalmol Sci. 2023;3:100318.
2. Vujosevic S, Alovisi C, Chakravarthy U. Epidemiology of geographic atrophy and its precursor features of intermediate age-related macular degeneration. Acta Ophthalmol. 2023;101:839–856.
3. Bressler SB, Muñoz B, Solomon SD, West SK, Salisbury Eye Evaluation (SEE) Study Team. Racial differences in the prevalence of age-related macular degeneration: The Salisbury Eye Evaluation (SEE) Project. Arch Ophthalmol. 2008;126:241–245.
4. Wong WL, Su X, Li X, et al. Global prevalence of age-related macular degeneration and disease burden projection for 2020 and 2040: a systematic review and meta-analysis. Lancet Glob Health. 2014;2:e106–116.
